# Protein Kinase C Activation Drives a Differentiation Program in an Oligodendroglial Precursor Model through the Modulation of Specific Biological Networks

**DOI:** 10.3390/ijms22105245

**Published:** 2021-05-15

**Authors:** Marina Damato, Tristan Cardon, Maxence Wisztorski, Isabelle Fournier, Damiana Pieragostino, Ilaria Cicalini, Michel Salzet, Daniele Vergara, Michele Maffia

**Affiliations:** 1 Department of Biological and Environmental Sciences and Technologies, University of Salento, 73100 Lecce, Italy; marina.damato@unisalento.it; 2 Laboratory of Clinical Proteomics, “Giovanni Paolo II” Hospital, 73100 ASL-Lecce, Italy; 3 Laboratoire Protéomique, Réponse Inflammatoire et Spectrométrie de Masse (PRISM), Université de Lille, INSERM, U1192, F-59000 Lille, France; tristan.cardon@univ-lille.fr (T.C.); maxence.wisztorski@univ-lille.fr (M.W.); isabelle.fournier@univ-lille.fr (I.F.); 4 Center for Advanced Studies and Technology (CAST), University “G. d’Annunzio” of Chieti-Pescara, 66100 Chieti, Italy; dpieragostino@unich.it (D.P.); ilaria.cicalini@unich.it (I.C.); 5 Department of Innovative Technologies in Medicine & Dentistry, University “G. d’Annunzio” of Chieti-Pescara, 66100 Chieti, Italy

**Keywords:** PKC, differentiation, oligodendrocytes, signaling, mass spectrometry, cytoskeleton, ROCK

## Abstract

Protein kinase C (PKC) activation induces cellular reprogramming and differentiation in various cell models. Although many effectors of PKC physiological actions have been elucidated, the molecular mechanisms regulating oligodendrocyte differentiation after PKC activation are still unclear. Here, we applied a liquid chromatography–mass spectrometry (LC–MS/MS) approach to provide a comprehensive analysis of the proteome expression changes in the MO3.13 oligodendroglial cell line after PKC activation. Our findings suggest that multiple networks that communicate and coordinate with each other may finally determine the fate of MO3.13 cells, thus identifying a modular and functional biological structure. In this work, we provide a detailed description of these networks and their participating components and interactions. Such assembly allows perturbing each module, thus describing its physiological significance in the differentiation program. We applied this approach by targeting the Rho-associated protein kinase (ROCK) in PKC-activated cells. Overall, our findings provide a resource for elucidating the PKC-mediated network modules that contribute to a more robust knowledge of the molecular dynamics leading to this cell fate transition.

## 1. Introduction

Specific molecular mechanisms control cellular identity in different biological systems through the involvement of internal and external factors. The intrinsic ability of a cell to respond to particular signals is defined as competence, and it is a necessary condition for cellular reprogramming. During these processes, plenty of stimuli from the microenvironment result in patterns of tissue-specific and stepwise changes in gene and protein expression, triggering the activation of lineage markers via interconnected crosstalk among multiple signaling pathways and nuclear transcriptional factors [1,2]. These molecular mechanisms represent potential targets for drug design and development, targeting and boosting strategic regulators of cell plasticity and reprogramming.

Several pieces of evidence support the involvement of protein kinase C (PKC) in the context of cell maturation and differentiation. PKC is best-known for triggering the differentiation of human U937 myeloid leukemia cells into immature macrophage-like cells [3]. PKC also plays a role in controlling epidermal differentiation and cutaneous homeostasis [4]. Moreover, it was demonstrated that N-methyl-D-aspartate receptors (NMDARs) stimulate the differentiation of multipotent juvenile sub-ventricular zone (SVZ) cells into oligodendrocytes through PKC activation [5]. As well as contributing to the normal physiological responses, including neuroprotection and cell cycle regulation [6], PKC is a central hub that controls a broad variety of downstream-signaling pathways associated with clinical disorders, including cancer [7] and neurodegenerative diseases, such as multiple sclerosis, Alzheimer and Parkinson [8,9,10]. The above evidence focuses the attention on PKC due to its central role in several physiopathological conditions and prompts us to investigate the mechanisms underlying PKC action in the context of oligodendrocyte differentiation.

In the central nervous system (CNS), remyelination primarily occurs from oligodendrocyte precursor cells (OPCs) that migrate to the lesion sites and differentiate into mature myelinating oligodendrocytes [11,12]. An enhanced oligodendrogenesis from OPCs has been observed after demyelination, suggesting that OPCs represent the major cellular targets to boost remyelination [13]. Genetic, epigenetic, and proteomic cues drive the oligodendrocytes differentiation. In this scenario, mass spectrometry (MS) has evolved in terms of sensitivity and specificity, thus representing a robust platform to analyze complex biological samples [14] and processes, which are studied as dynamic molecular networks [15]. MS approaches have been employed to establish a human oligodendrocytes (OLs) reference proteome database [16] and to define changes in protein expression during the process of OPCs maturation into OLs [17]. MS studies were also applied to define the heterogeneous nature of OPCs. For instance, Bribian and colleagues demonstrated that different metabolic patterns characterized OPCs in relation to their stage of maturity, thus affecting the proper design of pharmacological and cellular therapies for demyelinating diseases [18].

Here, we applied a label-free mass spectrometry (MS) approach to define the molecular response to PKC activation in the MO3.13 oligodendroglia cell model. In these cells, the activation of a differentiation program after PKC activation has been previously demonstrated [19,20,21,22,23], but, until now, never described at a comprehensive molecular level. We found that PKC activation drives a gliogenesis program associated with the modulation of other biological processes, including cytoskeletal remodeling, cell cycle and metabolism.

Overall, these results provide a resource for elucidating the PKC mediated signaling network able to lead a maturation process in MO3.13 cells.

## 2. Results

### 2.1. Phorbol 12-Myristate 13-Acetate (PMA) Treatment Induces Phospho-PKC Activation and Regulates MO3.13 Proliferation and Differentiation

**Figure 1:**
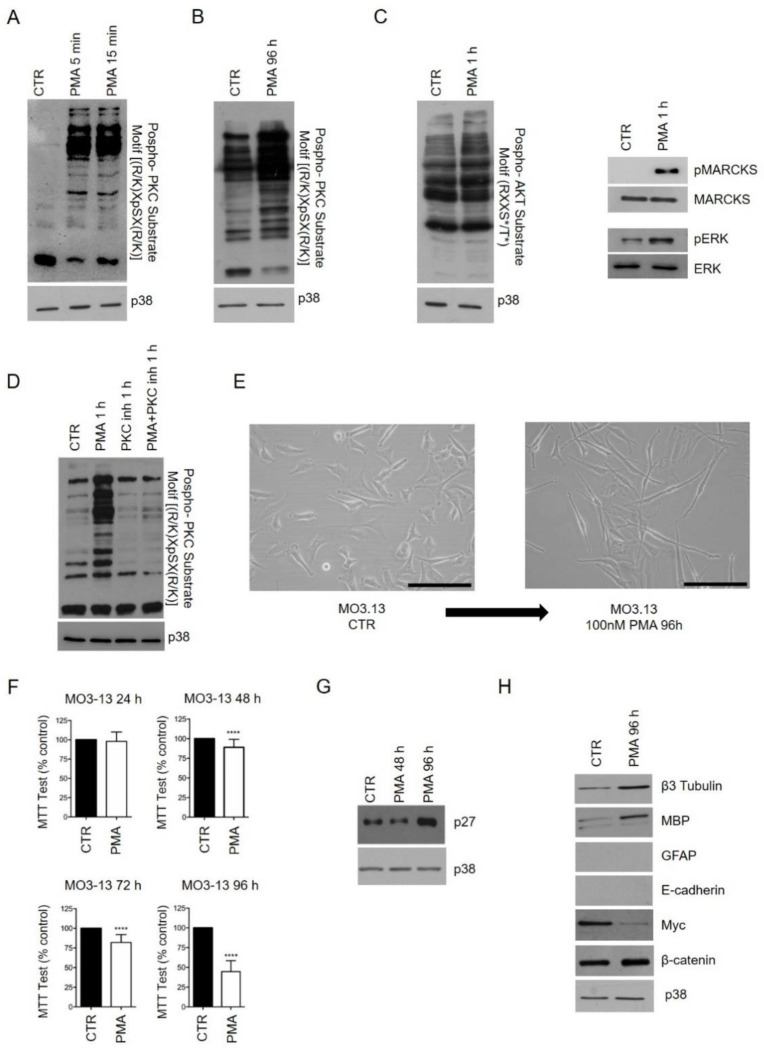
Phorbol 12-Myristate 13-Acetate (PMA) treatment induces phospho-protein kinase C (PKC) activation and MO3.13 differentiation. (**A**) Western blot analysis showing the activation rate of PKC substrates in MO3.13 cells after 100 nM PMA short-term treatment (5 min and 15 min). An anti-phospho-PKC substrate motif ((R/K)XpSX(R/K)) antibody was used. This antibody specifically detects endogenous levels of cellular proteins only when phosphorylated at Ser residues (S) surrounded by Arg (R) or Lys (K) at the ⁻2 and ⁺2 positions. p38 was used as a loading control. (**B**) Western blot analysis exhibiting the activation degree of PKC substrates after 96 h of PMA treatment compared to the control (CTR). p38 was used as a loading control. (**C**) Western blot analysis reporting the activation rate of AKT substrates, MARCKS and ERK proteins at 1 h of PMA treatment. (**D**) Western blot analysis reporting the activation of PKC substrates in MO3.13 cells, after treatment with PMA and Ro 31-8220 (1 μM) used alone and in combination for 1 h. p38 was used as a loading control. (**E**) Representative images of MO3.13 cells cultured in a normal condition medium (left) or treated with 100 nM PMA (right) for 96 h. Images were acquired using an inverted wide-field microscope (Olympus IX51). Scale bar 200 μm. (**F**) MO3.13 cell proliferation assessed by MTT test for 24 h, 48 h, 72 h and 96 h. Data were represented as the number of viable cells compared to the CTR, expressed as mean ± standard deviation (SD). **** *p*-value < 0.0001 by *t*-test. (**G**) Western blot analysis was performed on the whole-cell lysate using an anti-p27 antibody in MO3.13 cells treated with PMA for 48 h and 96 h. p38 was used as a loading control. (**H**) Western blot analyses using anti-β-tubulin3, anti-MBP, anti-GFAP, anti-E-cadherin, anti-Myc and anti-β-catenin antibodies were performed on the whole protein extract of MO3.13, CTR and treated conditions. p38 was used as a loading control. Western blot experiments were performed in triplicates.

To induce PKC activation, we stimulated MO3.13 cells with the diacylglycerol (DAG) analog PMA that acts by inducing the activation of DAG-sensitive PKC members. In accordance with previous reports, the molecule was used at the concentration of 100 nM for 96 h [19,20,21,22,23]. PMA induced rapid and sustained activation of PKC signaling, as revealed by Western blotting using a phospho-PKC substrate motif ((R/K)XpSX(R/K)) antibody (Figure 1A,B).

Furthermore, PMA treatment rapidly activated protein kinase B (AKT) substrates, myristoylated alanine-rich protein kinase C substrate (MARCKS) protein, the most prominent PKC cellular substrate, and extracellular-signal-regulated kinase (ERK) at 1 h (Figure 1C). Cotreatment of MO3.13 cells with PMA and PKC inhibitor Ro 31–8220 (1 µM) completely inhibited PKC activation (Figure 1D).

PMA induced marked changes in cell morphology. After 96 h, MO3.13-treated cells acquired an elongated phenotype, passing from a polygonal to a spindle-shaped morphology (Figure 1E). Substantial differences were also observed in the proliferation rate of control (CTR) and treated cells as determined by the MTT test (Figure 1F). In detail, PMA treatment led to a significant growth inhibition compared to CTR cells and, following this, a considerable upregulation of p27 protein was observed through Western blot after PMA treatment (Figure 1G). The overexpression of p27, a well-known cyclin-dependent kinase inhibitor, is associated with the negative regulation of cell cycle progression and positive cell differentiation regulation [24,25].

To determine whether PMA treatment was also associated with a modified expression profile of lineage markers and transcriptional factors (TFs) linked to the differentiation process, the whole protein extracts of MO3.13 cells, CTR and treated, were analyzed by Western blot (Figure 1H). Myelin basic protein (MBP), the major myelin component of the CNS associated with OLs, along with β-III-tubulin (TubβIII), a marker of neuronal lineage, both increased in the treated cells. On the contrary, the expression of other lineage markers (GFAP and E-cadherin) was not detected by Western blotting in CTR and treated cells, thus excluding any differentiation into mature astrocytes or epithelial cells. This finding was accompanied by changes in the expression of TFs, such as Myc and β-catenin proteins that, controlling cell proliferation and reprogramming, are also important for oligodendrocyte differentiation [26,27].

Overall, PMA induces a specific and rapid activation of PKC signaling, reduces the cellular proliferation rate and modulates the expression of lineage markers, thus driving a differentiation program in MO3.13 cells.

### 2.2. Networks Modulated after PMA Treatment

**Figure 2:**
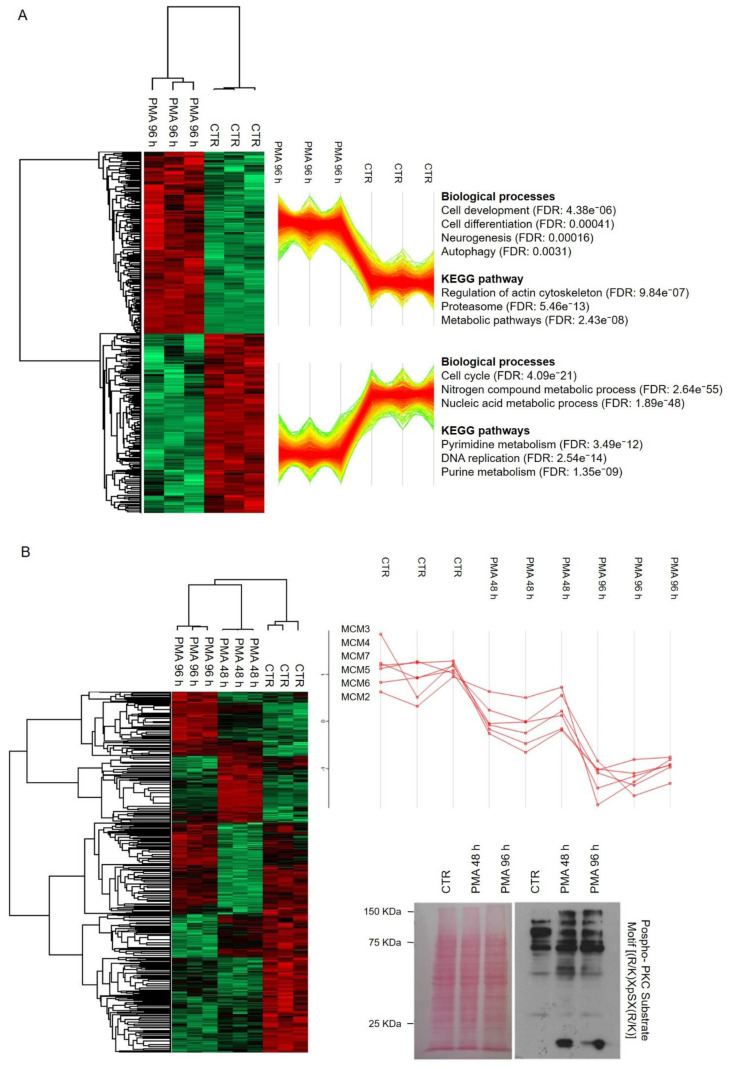

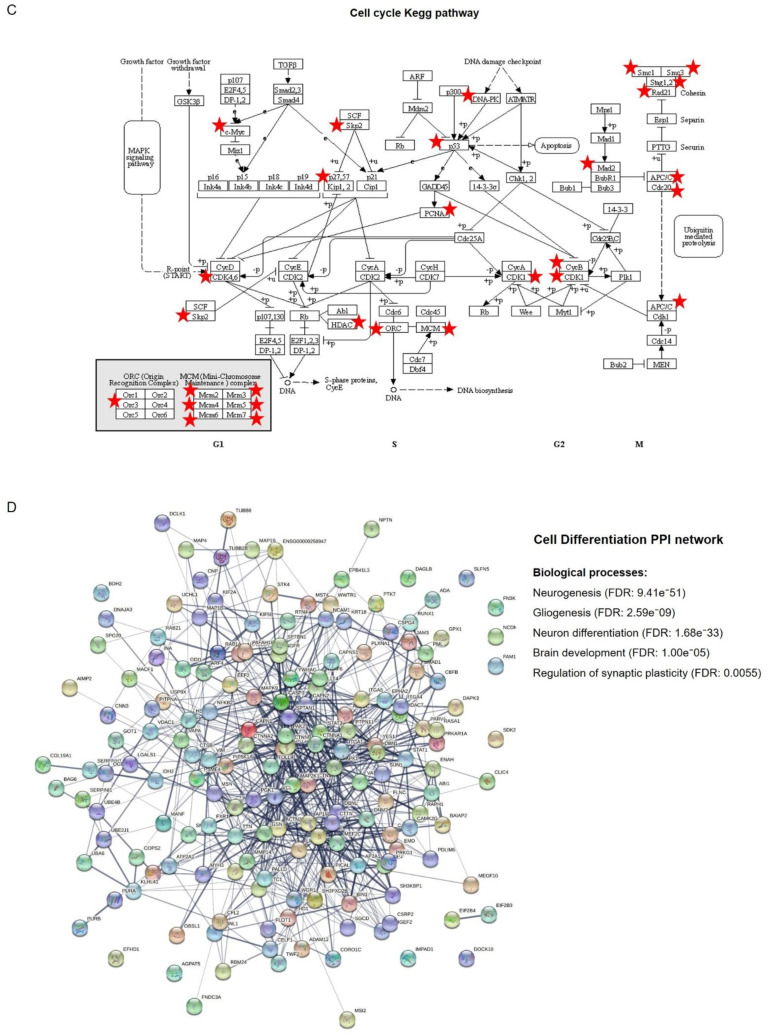
Networks modulated after PMA treatment. (**A**) Heat map based on Euclidean distance showing a significant separation between the CTR and the PMA treated conditions. Each row of the heat map represents a protein, and each column represents an independent sample. Two main clusters were identified from the hierarchical clustering, and their pattern is reported. (**B**) Heat map based on Euclidean distance showing proteome modifications occurring during MO3.13 treatment with PMA for 48 h and 96 h. The profile plot of the minichromosome maintenance complex proteins (MCM2-MCM7) is reported, and the time-dependent downregulated proteomic response to the treatment was significant at 96 h. Western blot analysis of PKC substrates activation after 48 h and 96 h of PMA treatment is reported. Blot stained for total protein with Ponceau S is reported. (**C**) Cell cycle KEGG pathway is visualized. The red stars indicate differentially expressed proteins involved in the pathway and identified by MS/MS, or Western blotting, after PMA treatment. (**D**) Protein–protein interaction (PPI) network analysis performed on the 175 proteins related to the statistically enriched biological process of “cell differentiation” (GO term) (FDR 0.00041), according to STRING software.

Next, we aimed to investigate more in detail the molecular changes associated with PKC activation. We took advantage of a label-free mass spectrometry high-throughput approach using proteins isolated from MO3.13 cells treated or not with PMA. More specifically, samples were lysed and trypsin-digested, as explained in the Materials and Methods section, and analyzed by high-performance liquid chromatography coupled to the tandem mass spectrometer (LC–MS/MS) Orbitrap Q-Exactive (Thermo Fisher, Waltham, MA, USA). After statistical validation using the Perseus software (1.6.2.1 version), 1465 unique proteins were identified as differentially expressed between CTR and PMA-treated cells (*p*-value < 0.05). In particular, the heat-map generated by Perseus segregated samples into two separated branches characterized by two clusters of up- and downregulated proteins (Figure 2A): 727 proteins resulted downregulated, while 738 proteins were upregulated after PMA treatment. Overall, this result highlights the large-scale proteome changes induced after PKC activation and supports the feasibility of our unbiased approach in revealing this molecular complexity.

The resulting MS dataset was then analyzed by the bioinformatics tool STRING 11.0 to perform a Gene Ontology (GO) analysis of enriched terms concerning biological processes, molecular functions and the Kyoto Encyclopedia of Genes and Genomes (KEGG) pathways (GO terms). GO analysis of downregulated proteins highlighted a statistical enrichment of proteins involved in biological processes related to cell cycle (FDR 4.09e⁻21), nitrogen compound metabolic process (FDR 2.64e⁻55), nucleic acid metabolic process (FDR 1.89e⁻48) and in KEGG pathways related to pyrimidine (FDR 3.49e⁻12) and purine metabolism (FDR 1.35e⁻09) and DNA replication (FDR 2.54e⁻14). It is well established the importance of cyclin-dependent kinase proteins (CDKs) in promoting transitions through the cell cycle and modulating transcription in response to specific signals [28]. Classical cell cycle CDKs, such as CDK1, CDK2, CDK6 and CDK9, as well as the regulatory protein G2/mitotic specific cyclin B1 (CCNB1), resulted significantly downregulated in our dataset. The cell-division cycle protein 20 (Cdc20) and members of the anaphase-promoting complex subunits (ANAPC7 and ANAPC4) also appeared statistically reduced. The downregulation of minichromosome maintenance complex proteins (MCM2–MCM7), which are involved in regulating DNA replication, was also observed in this group of proteins as similarly observed in other cellular models treated with PMA [29]. Proteomic analysis of the samples, treated for 48 h and 96 h with PMA, was performed, and the related heat map describes the experiment in Figure 2B. Profile plot of MCM family members, extracted from the dataset of differentially expressed proteins, reports the time-dependent downregulation of these proteins, reaching the maximum at 96 h (Figure 2B). A Western blot analysis confirmed the upregulation of phospho-PKC substrates at both time points (Figure 2B). Proteins related to the cell cycle KEGG pathway are visualized in Figure 2C.

Likewise, the cluster of overexpressed proteins after treatment was analyzed. Among the biological processes, cell development (FDR: 4.39e⁻06), cell differentiation (FDR: 0.00041), and neurogenesis (FDR: 0.00016) GO terms appeared to be statistically enriched. Other biological relevant pathways include the KEGG terms, proteasome (FDR: 5.46e⁻13), metabolic pathways (FDR: 2.43e⁻08) and regulation of actin cytoskeleton (FDR: 9.84e⁻07). In detail, the cell differentiation module proteins were mapped using STRING, showing a well-interconnected protein–protein network (PPI) that includes 175 members (Figure 2D). We hypothesized that a functionally important mediator of PKC differentiation signal could be found in this protein group. Supporting the functional significance of our precedent Western blot results, β-catenin occupies a central position in the PPI network, thus suggesting a possible central role in mediating PKC functional effects. This is consistent with the role of β-catenin signaling in expressing myelin genes, both in the peripheral Schwann cells and in oligodendrocytes [30]. Proteins identified in this network were associated with neurodevelopmental processes and categorized into neurogenesis (FDR: 9.41e⁻51), neuron differentiation (FDR: 1.68e⁻33), brain development (FDR: 1.00e⁻05), regulation of synaptic plasticity (FDR: 0.0055) and gliogenesis (FDR: 2.59e⁻09). In detail, the gliogenesis biological process included 16 members: CNP, CSPG4, CTNNB1, EEF2, EIF2B3, EIF2B4, GSN, MAP2K1, MAPK1, MMP14, PAFAH1B1, PTPN11, RTN4, STAT3, SUN1 and VIM. In particular, the overexpression of myelin protein 2′, 3′-cyclic-nucleotide 3′-phosphodiesterase (CNP) is responsible for dramatic changes in cellular morphology resulting in forming branched processes in OLs [31], and the actin-severing protein gelsolin (GSN) enhances actin dynamics and is essential for OPCs differentiation [32]. Tyrosine-protein phosphatase non-receptor type 11 (PTPN11 or Shp2) is required for OPCs generation and myelination process [33]. Considering other biological processes enhanced significantly in this dataset of proteins, we observed an enrichment of terms related to synaptic plasticity. This group includes BAIAP2, DBN1, MAPK1, MEF2C, NCDN, NPTN, and YWHAG proteins. Recently, a strict functional relationship between synaptogenesis and myelination has been provided, suggesting that signal mutations in synaptic genes could impair synaptic transmission and alter neuronal activity up to destroy oligodendrocyte maturation, myelination or survival [34]. This result could explain and provide functional support for the upregulation of synaptic proteins we observed after PKC activation.

### 2.3. Signaling Pathways Modulated by PKC Activation

**Figure 3:**
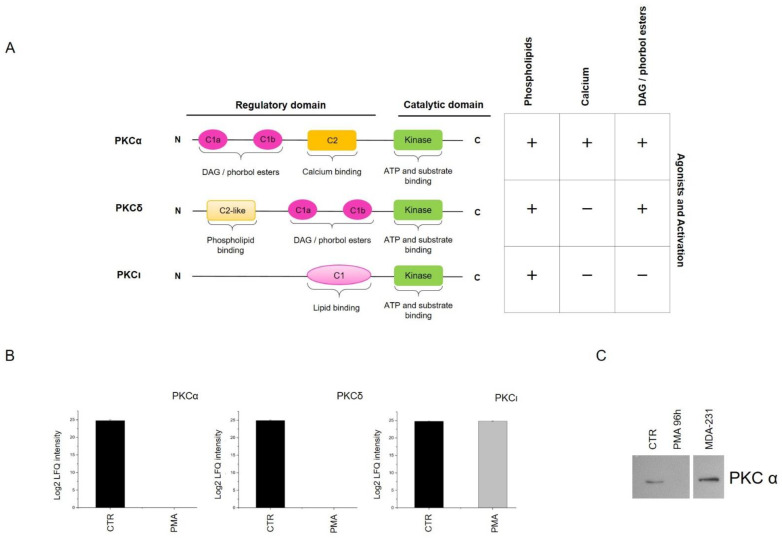
PKC isoforms and activated biological processes. (**A**) Schematic representation of structural conformation and agonists activation of PKCα, PKCδ and PKCι isoforms. (**B**) Histograms representing LFQ intensities of PKCα, PKCδ and PKCι isoforms, in logarithmic scale, for CTR and treated cells. (**C**) Western blotting showing the expression profile of PKCα isoform in CTR and treated cells. MDA-231 cell model was used as a positive control. Western blot experiment was performed in triplicates.

Based on their structures and activators, PKC isoforms are grouped into three subfamilies known as classical, novel and atypical (Figure 3A).

PKC activation involves a well-defined three steps process that includes maturation, activation and degradation of the activated isoforms. Once stimulated by PMA, classical and novel PKC isoforms are degraded mainly through a proteasomal pathway [35,36]. We searched for PKC isoforms in our MS/MS dataset and identified three different isoforms: PKCα, PKCδ and PKCι. We observed that PMA-activated isoforms PKCα (classical isoform) and PKCδ (novel isoform) were quantified only in CTR and were not detected in all PMA treated samples. On the contrary, the levels of PKCι were unaltered in PMA-treated cells compared to CTR (Figure 3B). We hypothesized that the prolonged exposure to PMA leads to the chronic activation of PKCα and PKCδ, thus activating a negative feedback loop leading to their degradation, as previously described. To confirm this, a significant downregulation of PKCα was observed by Western blotting in MO3.13-treated compared to untreated cells (Figure 3C).

To identify signaling processes downstream of PKC activation, we subjected to GO analysis differentially expressed proteins with a role in the regulation of signaling. From proteins upregulated after PMA treatment, we derived a list of 161 proteins that we further categorized into specific KEGG pathways. Regulation of actin cytoskeleton (FDR: 1.90e⁻06), cGMP-PKG-signaling pathway (FDR: 0.00014), MAPK-signaling pathway (FDR: 3.74e⁻05), hippo-signaling pathway (FDR: 0.00011), Focal adhesion (FDR: 0.00042), neurotrophin-signaling pathway (FDR: 0.00011), PI3K-Akt-signaling pathway (FDR: 0.0011), cAMP-signaling pathway (FDR: 0.0013), NF-kappa B-signaling pathway (FDR: 0.0195) and autophagy (FDR: 0.0031) emerged from this analysis.

Several studies have reported that autophagy’s lysosomal and recycling pathway promotes oligodendrocytes differentiation and survival due to its cytoprotective role [37,38,39]. In our dataset, LC3 (MAP1LC3B), a classic marker of the autophagic process, resulted upregulated (Supplementary Figure S1A). To confirm these data, Western blotting analysis was performed to test expressing p62. As expected, p62 resulted downregulated at 96 h of PMA treatment, compared to the CTR condition, supporting the hypothesis that autophagy is activated during the differentiation process (Supplementary Figure S1B).

**Figure 4:**
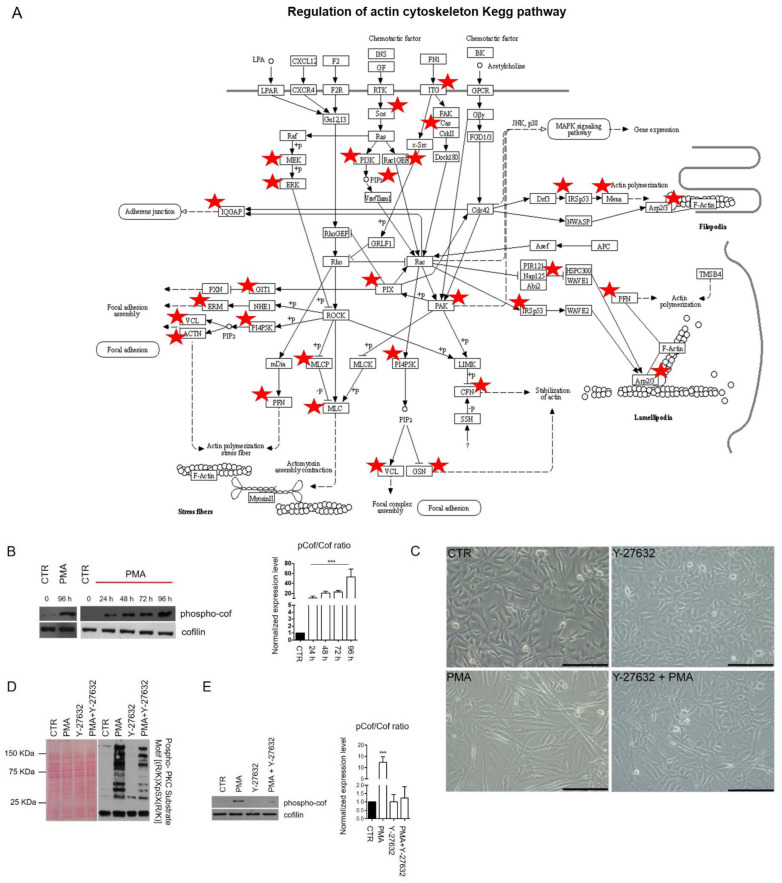

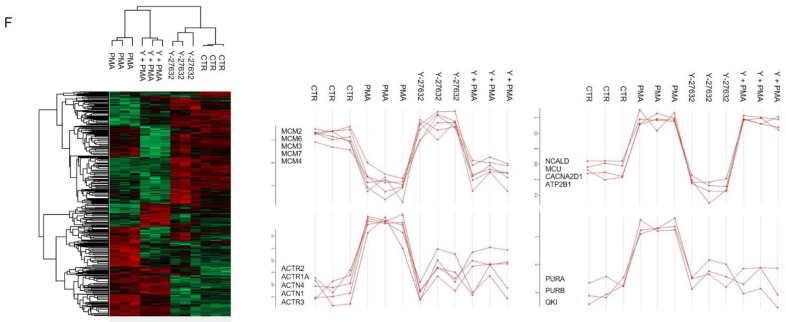
Signaling pathways and transcriptional factors modulated by PKC activation. (**A**) “Regulation of actin cytoskeleton” KEGG pathway is visualized. Red stars indicated differentially expressed proteins involved in the pathway and identified by MS/MS analysis, or Western blotting, after PMA treatment. (**B**) Expression profile of cofilin and phospho-cofilin was detected by Western blot at 0 h, 24 h, 48 h, 72 h and 96 h after treatment with PMA. Histogram represents the expression ratio of phospho-cofilin of the treated condition versus CTR sample. *** *p*-value < 0.001. (**C**) Phase-contrast images of MO3.13 cells cultured in a normal condition medium or treated with 100 nM PMA and 10 μM Y-27632, alone or in combination, for 96 h. Scale bar 200 μm. (**D**) Western blot analysis showing the activation level of PKC substrates in control and treated conditions after 1 h of stimulation. PMA at 100 nM and Y-27632 at 10 μM were used alone or in combination. The detection was performed through a phospho-PKC substrate motif ((R/K)XpSX(R/K)) antibody. The Ponceau S stained membrane is shown to verify the protein loading. (**E**) Western blot analysis showing the expression levels of cofilin and phospho-cofilin after treatment with PMA and Y-27632, used alone or in combination, for 96 h. Histogram represents the expression ratio of phospho-cofilin in the treated conditions versus the CTR sample. *** *p*-value < 0.001. (**F**) Hierarchical clustering based on Euclidean distance showing the separation between the CTR cells and those treated with PMA and Y-27632, alone and in combination. Profile plots of selected protein groups are shown, displaying distinct behavior concerning treatment.

Overall, this further supports a model in which multiple signaling networks cooperate to drive a differentiation program and identify biologically targetable components (i.e., network hubs) that are more likely to be functionally associated with the activation of specific PKC subnetworks. To test this idea, we examined the functional role of cytoskeleton proteins. Regulation of actin cytoskeleton resulted as one of the most significantly enriched pathways, and multiple studies support the role of the cytoskeleton in controlling cell differentiation and development [40,41]. The graphical representation of actin cytoskeleton KEGG pathway regulation is shown in Figure 4A.

In this figure, proteins identified by MS/MS and modulated after PMA treatment are shown using red stars. Rho-associated protein kinase (ROCK) protein lies at the center of this network, and several of our identified proteins are directly regulated by this kinase. To determine its possible activation status after PMA treatment, we evaluated the phosphorylation of cofilin, a downstream substrate of ROCK, in PMA-treated and untreated cells. Notably, phospho-cofilin levels, evaluated by Western blotting, significantly increased in a time-dependent manner after PMA stimulation, as shown in Figure 4B. Indeed, phospho-cofilin showed a > 50-fold upregulation after 96 h of PMA treatment. In a time-course experiment, we observed that cofilin phosphorylation was already detectable after 24 h of exposure to PMA (Figure 4B). Collectively, the results indicate an active role for ROCK in PMA treated cells.

To investigate whether cytoskeletal signaling mediated by ROCK affects PKC differentiation in MO3.13, we treated cells with the ROCK inhibitor Y-27643. We observed that PMA-induced morphological modifications of MO3.13 were reduced upon the combined treatment with PMA and Y-27632 (Figure 4C). Compared to the PMA treatment alone, the activation of PKC substrates resulted drastically reduced when PMA and Y-27632 were used simultaneously (Figure 4D). Moreover, the upregulation of phospho-cofilin was abrogated (Figure 4E), suggesting that phosphorylation of cofilin is regulated through the PKC/ROCK axis. To better characterize the effects of ROCK inhibition combined with PKC activation, we performed MS/MS analysis of MO3.13 cells treated with PMA and Y-26732 alone and drug combinations. Heat-map in Figure 4F shows a sample clusterization after ANOVA statistical analysis of the four groups. When cells were treated both with Y-27632 and PMA, we observed that ROCK inhibition affected specific PKC-enriched protein modules, reflecting the hypothesis that the ROCK pathway regulated particular biological processes. In detail, cell growth cycle and DNA replication biological processes were not influenced by ROCK inhibition. Consistent with this, MCM proteins were modulated in PMA and in PMA + Y-27632-treated samples, as shown in the profile plot of Figure 4F. In addition, proteins involved in calcium transport (NCALD, MCU, CACNA2D1, ATP2B1) were not modulated by the ROCK pathway. On the contrary, actin-related proteins, actinins (ACTR2, ACTR1A, ACTN4, ACTN1, ACTN3) and specific proteins regulating the expression of myelin protein components (PURA, PURB, QKI) resulted impaired after the combined treatment (Figure 4F). In particular, PURA and PURB transcriptional factors showed an increased expression in active myelinating CNS development [42]. Moreover, PURA has been demonstrated to regulate MBP gene transcription [43] and, under particular immunosuppressive conditions, into the demyelinating disease named progressive multifocal leukoencephalopathy, which precisely consists in the degeneration of the oligodendroglial cells [44]. QKI is an RNA-binding protein controlling the proper subcellular localization of MBP [45,46] and the splicing regulation of the myelin-associated glycoprotein (MAG) pre-mRNA [47]. The alteration of QKI expression may result in myelin disorders, such as multiple sclerosis and schizophrenia [48,49]. Recently, Zhou and collaborators highlighted the role of QKI in mature myelin preservation as a transcriptional regulator of genes involved in lipid metabolism. In mice, QKI depletion induced rapid demyelination and neurological disorders [50]. Overall, this finding identifies ROCK as a driver of a PKC module specifically involved in the control of cytoskeletal remodeling. In addition, we also found that the combined treatment with PMA and Y-27632 modulates expressing proteins involved in regulating myelin genes.

The hypothesis that cellular remodeling can regulate cell differentiation is consistent with the enrichment of the Hippo pathway members after PMA treatment. This pathway is involved in regulating many diverse biological processes, ranging from cell proliferation, cell fate determination and regeneration [51]. Specifically, CTNNA1, YWHAG, PPP2R1A, PPP2R2A, PPP1CA, CTNNB1, YWHAQ, PPP1CB, YWHAZ, CTNNA2, PPP2CA, WWTR1 (also known as TAZ) and SMAD1 were included among upregulated proteins. Western blotting analysis confirmed the overexpression of the main Hippo downstream effector YAP after 96 h of PMA treatment (Supplementary Figure S2).

This was consistent with the results of Shimizu and colleagues showing that the knockdown of YAP in OLs impaired their morphology and reduced the interaction with neurons in a co-culture system during myelination [52].

## 3. Discussion

Colonization of lesions by OPCs and their subsequent differentiation into mature myelinating oligodendrocytes are two essential events occurring during a remyelination process. Molecular mechanisms behind this transformation require a coordinated interplay among biological processes and signaling networks. The activation of PKC signaling drives a differentiation program in different cell types, including oligodendrocytes. For instance, Swire and colleagues demonstrated that PKCε promotes myelination in oligodendrocytes in vivo [53]; Cavaliere and collaborators found that subventricular zone cells differentiate into OLs through PKC activation [5]. Moreover, prior studies showed that PKC activation is an efficient strategy for reprogramming the precursor MO3.13 cells into mature oligodendrocytes [19,20,21,22,23]. An article recently published by Damiano and colleagues described the upregulation of OLs markers, such as MBP, after serotonin binding to a G-coupled protein receptor (5-HT2a) and the activation of PKC [23]. However, the molecular effectors of these events remain to be determined in detail. In this regard, there has never been reported high-throughput profiling effort to identify proteins modulated after PKC activation.

In this work, we performed a global investigation of PKC-induced proteome modifications in the human MO3.13 cell line. To dissect out the mechanism underlying the role of PKC on this maturation process, we performed an LC–MS/MS analysis of MO3.13 cells treated with the PKC activator PMA, obtaining a comprehensive characterization of the specific biological processes and molecular hubs that are modulated during the process of oligodendrocyte differentiation. Other works already applied MS to study the process of OL differentiation [18,54].

In MO3.13 cells, PKC stimulation induces rapid phosphorylation of PKC substrates. Specific PKC isoforms are potentially involved in mediating this effect. The expression of PKCα and PKCδ isoforms decreased after PMA treatment, in line with the mechanism of PKC regulation by the ubiquitin–proteasome pathway degradation. In several studies, the activation of these isoforms has been associated with the modulation of different processes, including the cell cycle. According to GO analysis, most of the proteins downregulated after PMA treatment are cell cycle components, consistent with previous studies [55]. The finding of Myc downregulation further supports this hypothesis as this protein represents a key transcriptional regulator of the transition from proliferating to differentiating OPCs [56]. Moreover, it has been asserted that, in several cell culture models of differentiation, Myc impairs this biological process maintaining the cells in a proliferative state; furthermore, Myc is dramatically downregulated when cells undergo terminal differentiation [57].

Gliogenesis and cytoskeletal remodeling have emerged as significant enriched biological processes after PKC activation. The gliogenesis program occurs in the developing and adult mammalian brain and consists of the production of glial progenitors and their differentiation into mature glia. Thus, overexpression of proteins involved in such a biological process clearly supports an ongoing differentiation program in the MO3.13 oligodendroglial cell model. In particular, CNP is a well-known abundant marker expressed in pre-myelinating and mature OLs, involved in RNA metabolism of highly myelinating regions and in OLs process formation [31]. Moreover, RTN4 is a protein extensively expressed in OLs, contributing to the differentiation of these cells [58], while Shp2 (or PPTN11) is a crucial controller of normal OPCs generation, differentiation and myelination, likely through MAPK-signaling regulation [33,59]. In this regard, MAP2K1 and MAPK1 are included in this upregulated network.

Cytoskeletal remodeling is also functionally associated with the process of differentiation [60,61], and the kinase ROCK leads at the center of this network. ROCK is broadly involved in cellular processes associated with differentiation and cellular reprogramming. For instance, it has been proven that the activation of Rho/ROCK signaling is responsible for the PMA-induced differentiation of U937 cells since the suppression of this pathway inhibits the maturation process [62]. Conversely, by inhibiting ROCK signaling, it is possible to induce terminal adipocyte trans-differentiation of the chemoresistant osteosarcoma AO cells [63]. Moreover, the pharmacological inactivation of ROCK in human keratinocytes resulted both in the inhibition of their terminal differentiation and in an increased proliferative capacity of these cells; this was in contrast with the effects of the constitutive expression of ROCK2 that led to cell cycle arrest and the expression increase of genes associated with terminal differentiation [64]. Furthermore, McBeath et al. discovered that inhibition of stress fibers formation led human mesenchymal stem cells towards an adipogenic fate, while the constitutive activation of ROCK and, consequently, stress fibers induced osteogenesis in the same cell line [65]. Moreover, in rat and human OPCs, Pedraza and colleagues demonstrated that knockdown of ROCK resulted in OPCs differentiation consisting in huge branches extension, increased and enhanced expression of myelin constituents [66]. All these data confirm that mechanical cues, such as cytoskeletal tension occurring during cell elongation and signaling pathways, are fundamental in the commitment of cell fate. To investigate the potential impact of ROCK on the protein networks orchestrated by PKC in the MO3.13 cell model, we tested if the inhibition of the kinase ROCK could interfere with the PKC-induced differentiation program. To do this, we inhibited the kinase ROCK using the Y-27632 inhibitor. Our data suggest a role for ROCK in the modulation of MO3.13 phenotype due to the reduced spindle-shaped morphology observed after treatment with PMA and the modulation of specific protein groups participating in the general process of differentiation and related to cytoskeletal and mechanotransduction dynamics. However, treatment with Y-27632, combined with PMA, is insufficient to block the activation of protein networks related to cell cycle regulation, DNA remodeling dynamics, calcium transport and neurogenesis, which are probably modulated by other pathways.

## 4. Materials and Methods

### 4.1. Cell Culture and Reagents

MO3.13 cells were purchased by Tebu-bio (www.tebu-bio.com, accessed date: 25 October 2016) and maintained in Dulbecco’s modified Eagle’s medium (4500 mg/L glucose, EuroClone, Milan, Italy) supplemented with 10% FBS, 100 U/mL penicillin, 100 µg/mL streptomycin and 2 mmol/L glutamine at 37 °C in an atmosphere of 5% CO2. To induce PKC activation, MO3.13 cells were daily stimulated with phorbol-12-myristate-13acetate (PMA) (Santa Cruz) at the concentration of 100 nM for 96 h.

### 4.2. Cell Viability Assay

Cells were seeded in a 96-well plate (5 × 10^3^/well) containing 100 μL of the complete medium in each well and let them adhere to the plate overnight. MTT (3-[4, 5-Dimethylthiazol-2-yl]-2, 5-diphenyltetrazolium bromide) test was performed according to the manufacturer’s instructions (SIGMA Aldrich, St. Louis, MO, USA). After reducing MTT to purple formazan in living cells, the absorbance of dimethyl sulfoxide (DMSO)-dissolved formazan crystals was measured at 570 nm using a microplate reader (iMark microplate absorbance reader, BIORAD). Experiments were performed in triplicates (*n* = 3) and repeated three times.

### 4.3. Sample Preparation and Mass Spectrometry Analysis

Whole protein extraction was carried out with the Illustra TriplePrep kit (GE Healthcare, Chicago, IL, USA) according to the manufacturer’s protocol. Subsequently, almost 20 μg of protein extract was processed according to the filter-aided sample preparation (FASP) protocol. Protein extract was dissolved and denatured in 8 M urea in 0.1 M Tris/HCl pH 8.5, reduced by an equivalent volume of 0.1 M DTT solution and heated at 56 °C for 40 min. The samples were loaded in Amicon Ultra 0.5 centrifugal filter device (Millipore) with a molecular weight cutoff of 10 kDa, washed three times with 8 M urea and centrifuged at 14,000× *g* for 30 min. This procedure was followed by the alkylation of the sample with 0.05 M of iodoacetamide solution for 20 min in the dark and centrifugation at 14,000× *g* for 30 min. After three washes with 8 M urea and three washes with 0.05 M NH4HCO3, the protein samples were digested by 20 μg/mL trypsin solution overnight at 37 °C. Each passage was followed by centrifugation at 14,000× *g* for 30 min. Peptides were collected by centrifugation followed by an additional wash with 0.5 M NaCl. Finally, the peptide mixture was acidified by 0.1% trifluoroacetic acid (TFA) solution, desalted-concentrated on C-18 ZipTip pipette tips from Merck Millipore (Merck KGaA, Darmstadt, Germany), eluted in 80% acetonitrile (ACN) and dried under vacuum. The sample was then resuspended in 20 μL of ACN/H20 (FA 0.1%) (98:2, *v/v*). Shotgun proteomics experiments were conducted in biological triplicates (*n* = 3).

### 4.4. Mass Spectrometry Analysis and Database Searching

The mass spectrometry analysis on the peptides was gained in reverse phase, using a chromatography system equipped with a pre-column (nano-Acquity Symmetry C18, 180 μm ID × 20 mm, 5 μm DP, Waters) to preconcentrate the peptides, and an analytical column (nano-Acquity BEA C18 column, 25 cm × 75 μm ID, 1.7 μm DP, Waters), used for their separation. Elution was carried out using a 2 h gradient of ACN/0.1% TFA starting from 5% to 30% for 120 min at a 300 nL/min flow rate. The chromatographic system was coupled with a Q-Exactive Orbitrap mass spectrometer (Thermo Scientific, Waltham, MA, USA) containing a nanoelectrospray ionization source. The analyzer was set with a resolution of 70,000 FWHM, an m/z mass range between 300–1600. An automatic gain control (AGC) of 3 × 10^6^ ions was used for a full MS scan and a maximum injection time of 120 ms. The minimum charge status of +2 was retained until +7 to exclude the unassigned load states, the +1 and >+8 charges. The MS/MS analysis was carried out, analyzing the most 10 intense ions within the primary MS study (top 10). The MS/MS fragmentation parameters were set at 17,500 FWHM, an m/z range between 200 and 2000, an AGC of 5E4 ions and a maximum time injection of 60 ms. MaxQuant proteomics software (version 1.6.1.0) [67] was used to analyze MS/MS raw files to match peptide sequences in the Human protein database from UniProt (release uniprot-human-reviewed-042018-20303seq.fasta), using Andromeda algorithm [68]. False discovery rate was set to 1% for peptides and proteins identification, and a minimum of 2 peptides per protein, with at least 1 unique, was defined. Label-free quantification of proteins was conducted using the MaxLFQ algorithm [69].

### 4.5. Experimental Design and Statistical Rationale

Shotgun proteomics experiments were conducted in biological triplicates (*n* = 3). Statistical analysis was performed with the Perseus software (version 1.6.2.1) [70]. Proteins identified in the decoy reverse database or only by site modification were not considered for data analysis. We also excluded the potential contaminants, and data were log2 transformed. Data were further filtered to ensure that identified proteins showed expression in at least two of the three biological samples for each considered condition. Missing values were replaced assuming a normal distribution, through the imputation function (downshift = 1.8; width = 0.3). Two-sample tests (*T*-test) or multiple sample tests (ANOVA) were performed (*p*-value = 0.05) to determine statistically significant differential protein expression. For hierarchical clustering, LFQ intensities were first z-scored and clustered using Euclidean as a distance measure for column and row clustering. Functional annotation and characterization of identified proteins were then performed using STRING version 11.0 (https://string-db.org, last accessed date 1 January 2021) [71].

Mass spectrometry data were deposited to the ProteomeXchange Consortium [72] via the PRIDE partner [73] repository with the dataset identifier PXD023336.

### 4.6. Western Blot Analysis

Cell lysates were extracted in RIPA buffer (Cell Signaling, Danvers, MA, USA) and quantified by the BRADFORD method (BIORAD, Hercules, CA, USA). About 25 μg of proteins were mixed 1:1 with Laemli buffer (SIGMA, St. Louis, MO, USA) boiled for 5 min, separated by 12% SDS–PAGE and transferred to the Hybond ECL nitrocellulose membrane (GE Healthcare, Chicago, IL, USA). Subsequently, membranes were blocked for 1 h in Blotto A (Santa Cruz, CA, USA) at room temperature and incubated for 1–2 h at room temperature with a primary antibody diluted in Blotto A. After two washes of 10 min with TBST solution (10 mM Tris, pH 8.0, 150 mM NaCl, 0.5% Tween-20), membranes were incubated with HRP-conjugated secondary antibodies for 2 h at room temperature. Membranes were washed twice for 5 min with TBST, and signals were then developed using the Amersham ECL Western blotting detection system (GE Healthcare, Chicago, IL, USA).

Membranes were probed with the following antibodies (1:1000 dilution): phospho-PKC substrate motif ((R/K)XPSX(R/K)) MultiMabTM (#6967), MARCKS (#5607), Phospho-MARCKS (Ser167/170) (8722), p38 (#9212), c-Myc (#13987), myelin basic protein (#78896), β-catenin (#8480), phospho-ERK1/2 (#4370), ERK1/2 (#4695), phospho-AKT substrate (RXXS*/T*) (#9614S), PKCα (#9960), SQSTM1/p62 (#39749) were from Cell Signaling. Phospho-cofilin Ser3 (sc-21867-R), p27 (sc-527), β3 tubulin (sc-80005), YAP (sc-101199) were from Santa Cruz Biotechnology. Cofilin 1 (10960-1-AP) was from Proteintech.

Secondary antibodies (HRP-conjugated) were from Santa Cruz Biotechnology (1:2000 dilution) (goat anti-mouse IgG-HRP, sc-2005; goat anti-rabbit IgG-HRP, sc-2004), or from Cell Signaling (1:2000 dilution) (anti-rabbit IgG, HRP-linked antibody #7074, anti-mouse IgG, HRP-linked antibody #7076). Densitometry analyses were performed using ImageJ (https://imagej.nih.gov, Access on 1 January 2021). Experiments were performed in triplicates (*n* = 3) and repeated three times.

## 5. Conclusions

Our data suggest a model in which the propagation of the signal from PKC, and its downstream target MARCKS, promotes MO3.13 differentiation via modulation of cell cycle processes, cytoskeletal remodeling, cell signaling, metabolism and neurodevelopmental processes. These results agree with the data of Schoor and colleagues, who demonstrated the enrichment of proteins related to cell cycle, nucleic acids metabolism, chromosome reorganization, cytoskeleton organization and lipid metabolism during primary rat OPCs differentiation into OLs [17].

The functional contribution of other molecular dynamics remains to be determined, and further experiments are needed to address this goal. For instance, we observed the modulation of metabolic processes after reprogramming with PMA, and an interesting direction would be to merge the proteome changes and the metabolomics.

## Data Availability

The mass spectrometry proteomics data were deposited to the ProteomeXchange Consortium via the PRIDE partner repository with the dataset identifier PXD023336.

## Supplementary Materials

The following are available online at https://www.mdpi.com/article/10.3390/ijms22105245/s1.
