# Supplementary material for: Protein Kinase C Activation Drives a Differentiation Program in an Oligodendroglial Precursor Model through the Modulation of Specific Biological Networks
Source: Int J Mol Sci. 2021 May 15;22(10):5245. doi: 10.3390/ijms22105245 (PMC8156399; doi:10.3390/ijms22105245)
Supplement: Supplementary file 1 [file ijms-22-05245-s001.zip › ijms-1170256-SI.pdf]

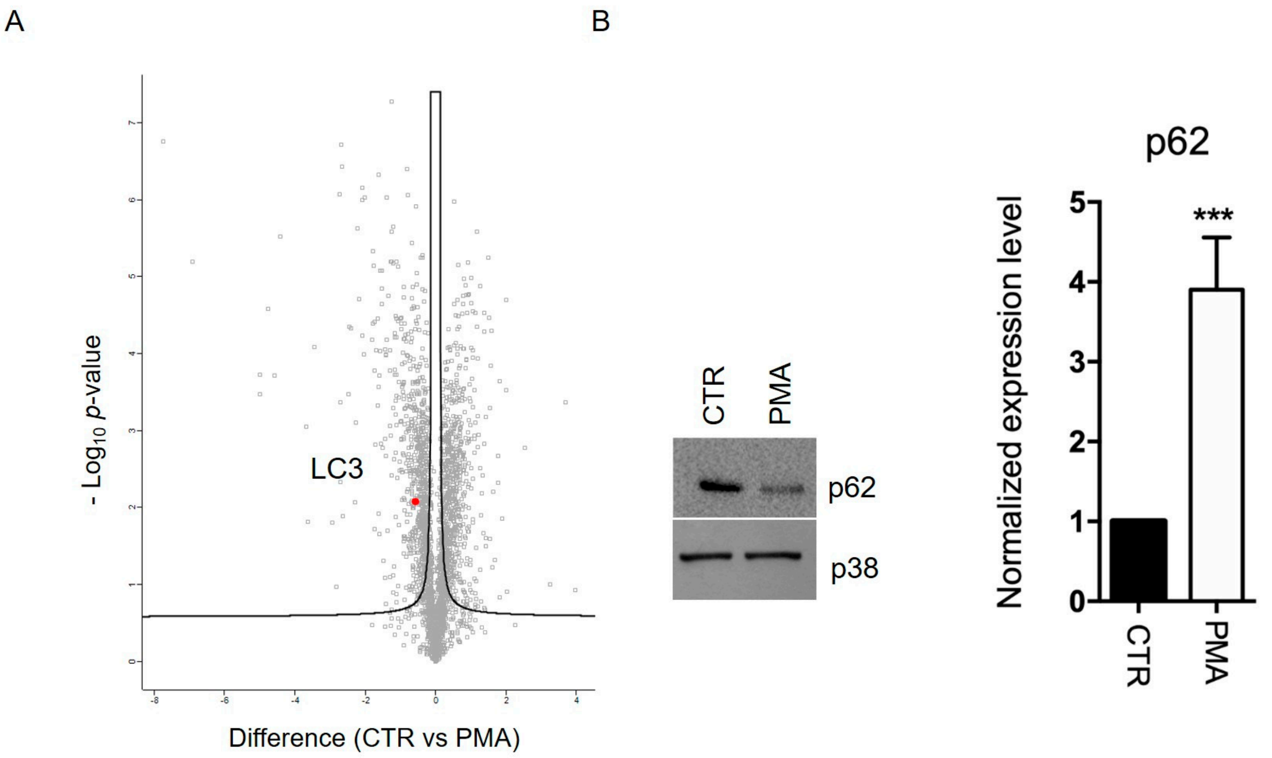

Supplementary Figure S1: PKC activation induces autophagy activation. A) Proteins obtained by MS/MS analysis of CTR and PMA treated cells are ranked in a volcano plot according to their statistical p-value (y-axis) and their difference (log2 fold change). LC3 is highlighted. The curve is derived at false discovery rate (FDR) = 0.05 and s0 = 0.1. **B)** Western blotting analysis showing the expression profile of p62 in CTR and treated samples. P38 was used as loading control. Western blotting experiment was performed in triplicates. Histogram represents the expression ratio of p62 in the treated versus the CTR sample. *** p-value < 0.001.

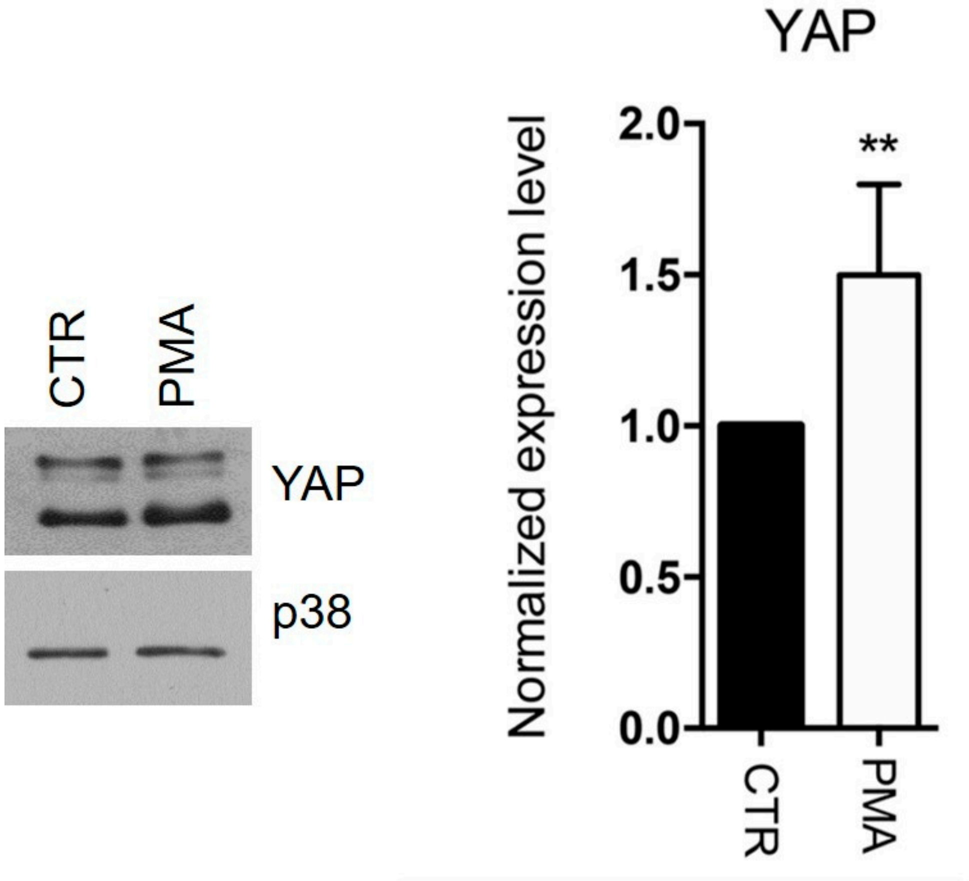

Supplementary Figure S2. PKC induces YAP over expression. Western blot analysis performed on the whole protein content of CTR and treated cells, using anti-YAP and anti-p38 antibody (loading control). Histogram represents the expression ratio of YAP in the treated versus the CTR sample. ** p-value < 0.01.
